# Long-Term Epidemiology and Evolution of Swine Influenza Viruses, Vietnam

**DOI:** 10.3201/eid2907.230165

**Published:** 2023-07

**Authors:** Jonathan Cheung, Anh Ngoc Bui, Sonia Younas, Kimberly M. Edwards, Huy Quang Nguyen, Ngoc Thi Pham, Vuong Nghia Bui, Malik Peiris, Vijaykrishna Dhanasekaran

**Affiliations:** The University of Hong Kong, Hong Kong, China (J. Cheung, S. Younas, K.M. Edwards, M. Peiris, V. Dhanasekaran);; National Institute of Veterinary Research, Hanoi, Vietnam (A.N. Bui, H.Q. Nguyen, N.T. Pham, V.N. Bui);; Centre for Immunology & Infection, Hong Kong (M. Peiris).

**Keywords:** Swine influenza virus, epidemiology, Vietnam, virological surveillance, serological surveillance, H1-δ1a virus, genome sequencing, phylogenetics, reassortment, risk assessment, influenza, viruses, respiratory infections, zoonoses

## Abstract

Influenza A viruses are a One Health threat because they can spill over between host populations, including among humans, swine, and birds. Surveillance of swine influenza virus in Hanoi, Vietnam, during 2013–2019 revealed gene pool enrichment from imported swine from Asia and North America and showed long-term maintenance, persistence, and reassortment of virus lineages. Genome sequencing showed continuous enrichment of H1 and H3 diversity through repeat introduction of human virus variants and swine influenza viruses endemic in other countries. In particular, the North American H1-δ1a strain, which has a triple-reassortant backbone that potentially results in increased human adaptation, emerged as a virus that could pose a zoonotic threat. Co-circulation of H1-δ1a viruses with other swine influenza virus genotypes raises concerns for both human and animal health.

Influenza A viruses (IAVs) pose a persistent One Health threat because of their ability to spill over into new host populations (*1*). Because pigs are susceptible to both avian and mammalian IAVs, they play an important role as mixing vessels in the generation of reassortant viruses (*2*). The 2009 influenza (H1N1) pandemic was caused by a reassortant H1N1 virus (pH1N1) containing gene segments from classical swine virus, Eurasian avian-like swine virus, and human seasonal virus lineages (*3*). Three different IAV subtypes (H1N1, H3N2, and H1N2) circulate in swine worldwide (*4*), with regional variation in antigenic characteristics. Contemporary swine influenza virus (swIV) lineages include H1N1, H1N2, and H3N2 triple-reassortant (TR) viruses that originated through reassortment of classical swine, avian, and human influenza viruses in the 1990s (*1*,*5*), European avian-like H1N1 swIV introduced into pigs during the 1970s, and several human H1N1- and H3N2-derived viruses, including pH1N1, pre-2009 H1N1, and H3N2-variant viruses (*4*). The increasing genetic diversity of IAVs in swine presents a pandemic concern (*4*,*6*). Human infections of emerging swIV are most frequently reported in the United States, where all nonhuman influenza viruses are nationally notifiable, with recurring outbreaks at agricultural fairs (*7*,*8*); however, sporadic human cases due to swIVs have been recently reported in Asia (*9*,*10*), Europe (*11*), and Australia (*12*).

In 2013, swIV surveillance was established at a collective slaughterhouse in Hanoi, Vietnam, that sourced pigs from 23 local provinces (*13*) (Table 1). During 2013 and 2014, cocirculation of multiple swIVs was detected, including viruses originating from pH1N1, H1N2 (with pre-2009 seasonal influenza–derived H1 hemagglutinin [HA]), H3N2 derived from human seasonal influenza, and TR H1N2 and H3N2 viruses (*13*). For this study, we extended virologic surveillance to 2019 and serologic surveillance from 2013 to 2019 in this slaughterhouse to characterize swIV evolution. 

**Table 1:** Summary of swine influenza virus isolates, by province, from samples collected at Van Phuc slaughterhouse in Vietnam during 2010–2019*

Province of origin	No. samples	No. (%) positive
Ha Noi	2,178	52 (2.39)†
Ha Nam	855	10 (1.17)
Hai Phong	61	7 (11.48)
Bac Ninh	51	4 (7.84)
Thai Nguyen	199	3 (1.51)
Ninh Binh	22	1 (4.55)
Nghe An	51	1 (1.96)
Hung Yen	96	1 (1.04)
Vinh Phuc	215	1 (0.47)
Hoa Binh	465	2 (0.43)
Phu Tho	301	1 (0.33)
Bac Giang	200	0
Yen Bai	150	0
Thanh Hoa	45	0
Thua Thien Hue	31	0
Nam Dinh	29	0
Dien Bien	2	0
Thai Binh	135	0
Tuyen Quang	27	0
Dong Nai, SV	195	11 (5.64)
Binh Duong, SV	16	0
Binh Dinh, SV	10	0
Kieu Ninh, SV	7	0
Location not specified	509	20 (3.93)
Total	5,850	114 (1.95)†

*SV, southern Vietnam.  †Includes 1 mixed infection of H1N2 and H3N2.

## Materials and Methods

### Surveillance and Virus Isolation

We examined 150 paired serum and nasal swab specimens collected from monthly convenience sampling at the Van Phuc slaughterhouse, the main collective swine slaughterhouse in Hanoi, Vietnam. We recorded the province of origin for each sampled pig. We report, in this study, findings from serum samples collected May 7, 2013–August 28, 2019. Virus isolation and sequence data from swab samples collected May 7, 2013–May 19, 2016, were reported previously (*13*); virus isolation and sequence data from swab samples collected June 30, 2016–August 28, 2019, are reported in this study. We cultured samples for virus isolation in MDCK cells at the National Institute of Veterinary Research Laboratory in Hanoi using described methods (*13*).

### Whole-Genome Sequencing and Assembly

We amplified the influenza genome from RNA extracted from virus isolates using a multisegment reverse transcription PCR (RT-PCR) with universal primers (*14*,*15*) and sequenced by using Nextera DNA library preparation and MiSeq (PE300) sequencer (Illumina, https://www.illumina.com), as previously described (*13*). We removed adapters and low-quality reads by using Trimmomatic version 0.36 (USADEL LAB, http://www.usadellab.org), and we performed de novo assembly by using IVA version 0.8.1 (*16*). We mapped the assembled contiguous sequences to the reference sequence using Geneious Prime version 2019.2.1 (Geneious, https://www.geneious.com) and generated consensus sequences by merging overlapping contigs. We submitted to GISAID (https://www.gisaid.org) the Vietnam swIV genome sequences and associated metadata (Appendix 1). We used BLAST (https://blast.ncbi.nlm.nih.gov/Blast.cgi) to analyze sequences generated in this study with other closely related influenza viruses from the GenBank and GISAID databases, including vaccine strains of human seasonal influenza H1N1. We classified H1 clades according to a phylogeny-based global nomenclature system (*17*).

### Phylogenetic Analysis and Genotypic Diversity

We aligned gene segments individually by using MAFFT version 7.490 (*18*) and constructed maximum-likelihood phylogenetic trees by using IQ-TREE version 2.1.4 on the basis of the best-fit nucleotide substitution model determined by ModelFinder (IQ-TREE, http://www.iqtree.org), estimating branch supports by using an approximate likelihood ratio (SH-like) test (*19**)*. We determined genotypes of swIVs by assigning each segment to specific lineages on the basis of maximum-likelihood phylogenies and characterizing the genotype based on the clade distribution of its internal segments (*20*). We inferred time-scaled phylogenies calibrated by sample collection dates by using the maximum-likelihood method in IQ-TREE version 2.1.4, implementing a least-square dating algorithm (*21*). We visualized trees in FigTree version 1.4.4. (http://tree.bio.ed.ac.uk/software/figtree).

### Serologic Assays

We randomly selected 10 pig serum samples from each monthly sampling visit to the same slaughterhouse (*13*). In total, we tested 760 pig serum samples collected during May 2013–August 2019 by using the hemagglutination inhibition (HI) assay against 3 swine H1 subtype influenza virus strains and 1 swine H3-subtype influenza virus strain isolated in this study, as well as pH1N1 virus. Those viruses were A/California/04/2009 (pH1N1), A/swine/Hanoi/7-305/2016 (H1-TR, H1N2), A/swine/Hanoi/11-260/2019 (H1-like, H1N2), A/swine/Hanoi/12-276/2019 (H1-δ1a, H1N2) and A/swine/Hanoi/10-984/2018 (2004/05 human H3N2-origin). We grew the viruses in MDCK cells and used them as antigens. The HI tests were carried out according to the World Health Organization’s standard protocol for animal influenza diagnosis (*22*), with details as previously described (*13*).

## Results

### Incidence of swIVs and Subtype Diversity

**Figure 1:**
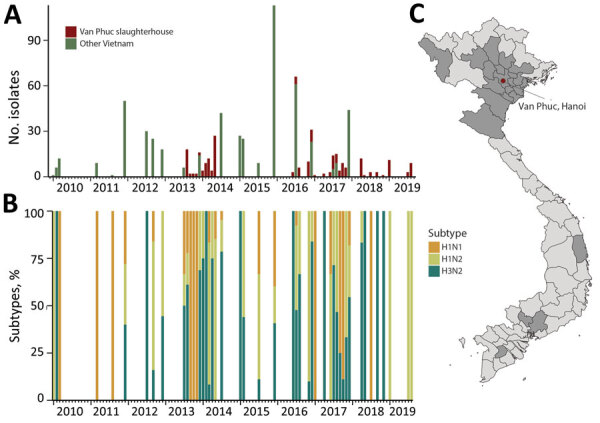
Swine influenza virus detection in Vietnam during 2010–2019. A) Number of swine Influenza viruses isolated from Van Phuc slaughterhouse in Hanoi during 2013–2014 (*13*,*26*) and 2016–2019, alongside other surveillance studies in Vietnam (*23*,*27*,*28*). B) Percentages of swine influenza virus subtypes detected. C) Provincial origins (gray shading) of pigs sampled at Van Phuc slaughterhouse during 2016–2019.

No clear pattern of seasonality was detected from the 150 paired swab and serum specimens collected during June 2016–August 2019, nor from previous studies conducted during 2010–2016 (Figure 1, panels A, B). The abattoir sourced swine from 23 provinces in Vietnam (Figure 1, panel C); most samples (87%) originated from provinces in northern Vietnam, 4% of samples originated from southern provinces, and location data were missing for the remaining samples (9%). Most swIV-positive samples originated from Hanoi (46%), Ha Nam (9%), and Hai Phong (6%) in northern Vietnam and Dong Nai (10%) in southern Vietnam (Table 1; Figure 1, panel C). There were 114 influenza-positive nasal swab samples with 1 mixed-subtype infection among the 5,850 swabs tested, yielding 115 viruses for study, for an isolation rate of 1.95%.

Subtyping of the 115 swIV isolates showed the circulation of 3 virus subtypes, H1N1 (n = 16), H1N2 (n = 46), and H3N2 (n = 53); 1 H1N2 and H3N2 co-infection was detected. Our previous study in the same abattoir during 2013–2014 yielded the same 3 virus subtypes, H1N1 (n = 16), H1N2 (n = 35), and H3N2 (n = 26) (*13*). Of note, we observed a high genetic similarity of viruses collected during each sampling at the Van Phuc slaughterhouse, suggesting that viruses were not maintained in the abattoir but were repeatedly introduced from sourced populations.

### Phylogenetic Relationships of the HA Genes

**Figure 2:**
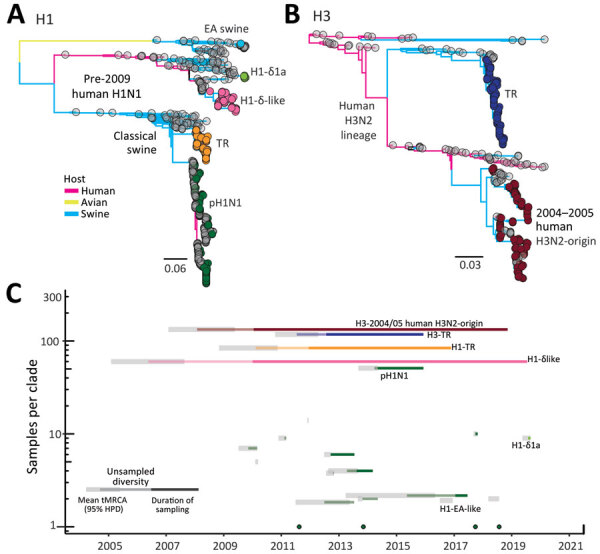
Genomic epidemiology of swine influenza viruses in Vietnam. A, B) Maximum-likelihood phylogeny of the H1 (A) and H3 (B) hemagglutinin genes of swine influenza viruses. Branch tips are colored by lineage origin and branches by host. C) Persistence of independent hemagglutinin lineages of swine influenza viruses detected in Vietnam. EA, Eurasian avian; HPD, highest posterior density; tMRCA, time to most recent common ancestor; TR, triple reassortant.

We conducted a maximum-likelihood phylogenetic analysis of the HA genes of 145 swIVs collected during 2016–2019 in Vietnam, together with virus sequences from previous studies from the same abattoir during 2013–2019 and 390 swIVs collected in Vietnam by other studies (Figure 2, panel A). HA sequence data revealed the circulation of 4 distinct H1 lineages and 2 distinct H3 lineages over various time points (Figure 2, panel B; Appendix 2 Figure 1). The H1 subtype viruses included human-derived pH1N1 (n = 122), classical swine-derived H1-TR (n = 84), and pre-2009 seasonal H1N1 (n = 69), which we further classified into H1-δ–like (n = 60) and H1-δ1a (n = 9). In addition, 2 viruses of the H1N2 subtype collected in December 2016 by Takemae et al. (*23*) belong to the Eurasian avian-like swine H1 HA lineage. The H3-HA phylogeny indicates swIVs in Vietnam belong to either the swine-origin North American TR lineage or the 2003/04 human H3N2-origin swIV first detected in Vietnam in 2010 (Figure 2, panel B; Appendix 2 Figure 1).

HA phylogenies identified the circulation of 21 independent transmission lineages in swine in Vietnam since 2010, including 4 singletons (Figure 2, panel B). Those lineages were all identified elsewhere in the world and were likely introduced by imported swine or by humans through reverse zoonosis. Of the 21 transmission lineages, 17 independent transmission lineages of pH1N1 virus were detected at various time points. Most introductions were limited to 1–14 samples collected during 1–3 contiguous sampling months and were most closely related to spillover of contemporary human pH1N1 viruses. Two closely related pH1N1 reassortants detected in January and June 2017, A/swine/Hanoi/7_619/2017 and A/swine/Hanoi/8_463/2017, derived HA and neuraminidase (NA) genes from humans around 2013 and polymerase basic (PB) 2, PB1, or nonstructural genes from TR swIVs from China. Long branches in the HA and NA phylogenetic trees suggest circulation in swine for >3 years.

**Figure 3:**
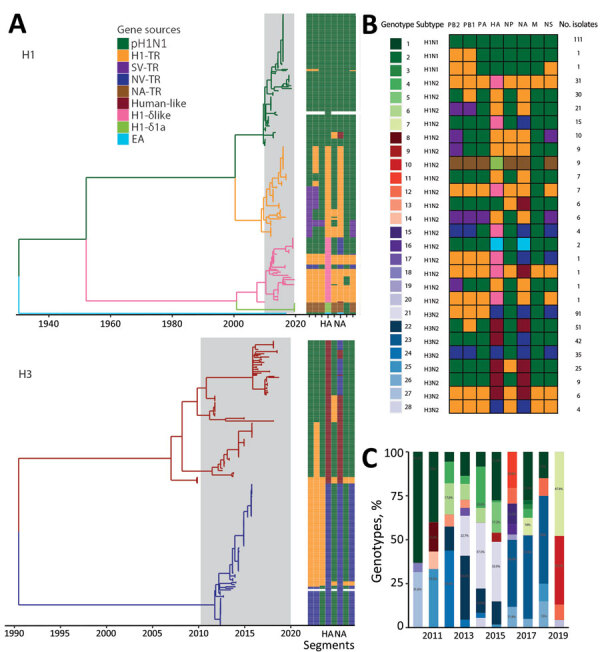
H1 and H3 swine influenza virus genotypes in Vietnam, 2010–2019. A) Temporal phylogeny of HA genes. Branches are colored by lineage of origin. Heatmap illustrates gene segment origins. B) Genotypes identified in Vietnam during 2010–2019. Box shading denotes lineage of origin by color grouping, as shown in panel A. C) Annual percentages of genotypes identified. Colors are as shown in panel B. C-TR, Chinese triple reassortant; EA, Eurasian avian; H1-δ, pre-2009-human H1-δ; H1-δ1a, pre-2009 human H1-δ; HA, hemagglutinin; M, matrix; NA, neuraminidase; NA-TR, North American triple reassortant; NP, nucleoprotein; NS, nonstructural; NV-TR, northern Vietnam triple reassortant; PA, polymerase acidic; PB, polymerase basic; SV-TR, southern Vietnam triple reassortant.

In addition to recurrent detections of pH1N1 during 2014–2017 (n = 53), a single lineage of H1 TR virus (n = 84), closely related to swIV in Guangxi Province, China, during 2010–2011, was maintained during 2010–2019, and an H1-δ-like lineage (n = 60) was derived independently from pre-2009 human seasonal H1N1 viruses that circulated in 2004–2006. Nine virus sequences collected on the last sampling occasion (in August 2019) belonged to the H1-δ1a lineage and were most closely related to H1N2 swIVs circulating in the United States during 2015–2016 (Appendix 2 Figure 3). The H1-δ1a originated from human seasonal H1N1 viruses circulating in humans during 2002 and 2003 (*24*,*25*) and was previously found only in swine in the United States (*17*).

Two major H3-HA lineages originated from North America. One belongs to the TR lineage that circulated during 2012–2015 in northern Vietnam (n = 132) (*13*,*26**,**27*), and the other belongs to the 2004/05 human H3N2-origin swIV (n = 133) that circulated during 2010–2018 (Figure 2, panel B). The 2004/05 human H3N2-origin lineage clustered with viruses circulating in southern China and Cambodia during 2011–2012 and a zoonotic case reported in Vietnam (A/Ho Chi Minh/459-6/2010 [H3N2]). The 2004/05 human H3N2-origin viruses have been further classified into 2 divergent lineages, NV and SV, based on detection in north and south Vietnam. SV viruses have not been detected since 2015, and NV viruses were detected up to 2018. Taken together, our results indicate a recent bottleneck in overall HA diversity; H1 and H3 TR lineages have not been detected since 2016, and detections of new human pH1N1 viruses have been declining. However, H1-δ viruses became predominant by 2018 and 2019.

Temporal phylogenies of the H1 and H3 HA genes of Vietnam swIVs (Figure 2, panel B; Figure 3, panel A) show long branches of unsampled diversity leading up to the H1-δ–like virus and the 2004/05 human H3N2-origin swIV, which was before initiation of swine surveillance in Vietnam. The pH1N1 virus lineage we described circulated for ≈4 years before detection, and within the swIV clades from Vietnam, long branches of unsampled diversity span 4 to 5 years. A sublineage of the 2004/05 human H3N2-origin virus that diverged before 2010 was only detected in 2016–2019. Similarly, recent viruses belonging to 2 H1-δ sublineages appear to have been sampled after 4 years.

### Genotypes of swIV in Vietnam during 2010–2019

Phylogenetic analysis of each gene segment from 2010–2019 showed that the swIV gene pool in Vietnam includes genes from pH1N1 virus, pre-2009 human-derived H1 viruses (further subdivided into H1-δ–like and H1-δ1a), Eurasian avian-like H1 virus, and TR viruses of H1 and H3 subtype (Figure 3). Of the combined 537 swIVs, 111 H1N1 swIVs derived entirely from pH1N1 (genotype 1), whereas 2 H1N1 (genotypes 2–3), 161 H1N2 (genotypes 4–20), and 263 H3N2 (genotypes 21–28) viruses were reassortants (Figure 3). Among the 426 reassortant H1N2 and H3N2 swIVs, we detected several preferred genetic constellations (Figure 3, panel B). We identified 28 swIV gene constellations, designated as genotypes 1–28, from the 2010 to 2019 time frame, of which 17 were previously reported (*13*,*26**-**28*). Of those, only genotypes 1, 4, and 25 continued to be detected during 2016–2019. Genotypes 1 and 4 were detected in both northern and southern Vietnam; the others were found in either northern or southern Vietnam (Figure 3; Appendix 2 Figure 1).

In each year, we observed the cocirculation of multiple genotypes; few genotypes were maintained across multiple years (Figure 3, panel C). Most of the swIVs detected in Vietnam were genotype 1, which had all 8 gene segments originating from human pH1N1 viruses (n = 111) (Figure 3; Appendix 2 Figure 1). Genotype 1 was dominant in Vietnam during 2010–2015 and 2017–2018, followed by genotypes 23, 7, 24, and 10, found during 2010–2019 (Figure 3, panel C). We found that pH1N1 viruses with PB2, PB1, nucleoprotein (NP), and nonstructural genes from TR viruses and N2-NA genes from human H3N2-derived swIV (genotypes 2, 3, and 13) were detected sporadically (Figure 3). The NA and internal genes of swine pH1N1 viruses were not monophyletic but interspersed between human-origin virus sequences. All potential spillover events were closely related to human viruses, not limited to human viruses in Vietnam.

In contrast to the predominance of a single pH1N1 virus genotype, the H1N2 TR viruses repeatedly acquired internal genes from pH1N1 viruses (genotypes 5, 6, 8, 9, and 14); the most recent H1N2 TR viruses contained all internal genes of pH1N1 viruses. Two H1N2 swIV sequences (genotype 16) contain Eurasian avian-like surface proteins and pH1N1 internal genes. Similarly, the genotype 8 H1N2 viruses, with H1-δ HAs and all other segments from TR viruses, repeatedly gained pH1N1 genes to form genotypes 7, 12, 15, and 20. The most recent H1N2 TR viruses collected in 2019 contain TR N2 and pH1N1 internal segments (genotype 7). However, H1-δ genotype 4 viruses with no pH1N1 genes continued to be detected until 2019. Furthermore, H1-δ1a viruses (genotype 10) isolated in August 2019 contained North American TR internal and NA genes with only the matrix protein (M) gene from pH1N1. Those viruses have not been seen anywhere other than the United States and now Vietnam, suggesting that those swIVs likely spread to Vietnam directly from the United States.

We detected 8 distinct H3N2 swIV constellations (genotypes 21–28). The H3 TR viruses that circulated in northern Vietnam during 2012–2015 were initially detected with all genes of TR lineage (genotype 28) or with polymerase acidic, NP, and M genes derived from pH1N1 viruses (genotype 24). However, H3 TR viruses detected during 2014–2015 contained only NP and M genes from pH1N1 (genotype 21). The 2004/05 human H3N2-derived viruses, initially identified in 2010 with all internal genes from TR lineages (genotype 27), gained pH1N1 gene segments with or without maintaining TR PB1 (genotype 22) or NP (genotype 25) genes. However, the most recent viruses of this H3 lineage (detected in 2017–2018) contained the TR N2 and pH1N1 internal genes (genotype 23). Taken together, our findings show that swIV lineages of all subtypes collected in recent years in Vietnam have acquired the pH1N1 internal genes, except 2 divergent H1-δ viruses (genotype 4 and 10) that appear to have become predominant in Vietnam in recent years.

### Seroprevalence of swIV in Northern Vietnam

Of swine serum samples tested by HI assay, 41% (309/760) showed antibody titers ≥40 against at least 1 of 5 representative virus antigens tested (Table 2). Seroprevalence of 2004/05 human H3N2-origin virus in 2016–2019 (15.3%) was broadly comparable to that for 2013–2014 (15.9%), whereas H1 lineage seroprevalence varied over time. Seroprevalence of pH1N1 and H1-TR virus decreased, and H1-δ–like virus seroprevalence increased from 13% in 2013–2014 to 20% in 2016–2019. H1-δ1a antibodies were only detected in 2016–2019; the first seropositive samples were collected in March 2016 (Appendix 2 Table 1) and showed limited cross-reactivity to other H1 swIV lineages (Appendix 2 Table 2) (*29**,**30*). Despite continuous detection of an H1-TR lineage during 2013–2017 (Figure 2, panel B), overall seropositivity against the H1-TR reference strain, A/swine/Hanoi/7_305/2016(H1N2), remained low at 2.4% (Table 2). H1-TR seropositive samples displayed elevated HI titers against pH1N1 (2- to 3-fold increase; Appendix 2 Table 3). This result suggests H1-TR reactive antibodies are likely the result of cross-reactivity with pH1N1.

**Table 2:** Seroprevalence against representative swine influenza A viruses isolated from Van Phuc slaughterhouse in Vietnam, 2010–2019*

HA lineage	Reference antigen	No. (%) seropositive†		
		2013–2015, n = 320	2016–2019, n = 440	Overall, n = 760
2009 pandemic H1N1	A/California/04/2009 (H1N1)	57 (17.8)	61 (13.9)	118 (15.5)†
Pre-2009 human seasonal H1-δ–like	A/swine/Hanoi/11-260/2019 (H1N2)	43 (13.4)	89 (20.2)	132 (17.4)†
Pre-2009 human seasonal H1-δ1a	A/swine/Hanoi/12-276/2019 (H1N2)	0	36 (8.2)	36 (4.7)
H1-TR	A/swine/Hanoi/7-305/2016 (H1N2)	10 (3.1)	8 (1.8)	18 (2.4)
2004/05 human H3N2-origin	A/swine/Hanoi/10-984/2018 (H3N2)	49 (15.3)	70 (15.9)	119 (15.7)
Total positive		126 (39.4)	183 (41.6)	309 (40.7)

*Seropositivity defined as hemagglutination inhibition assay titer ≥40. HA, hemagglutinin; TR, triple reassortant. †A total of 30 serum samples were positive for both A/California/04/2009 (H1N1) and A/swine/Hanoi/11–260/2019 (H1N2). Because cross reactivity between these viruses is low, this result likely indicates sequential infections.

## Discussion

Our study, conducted during 2016–2019, provides insights into the evolution and epidemiology of swIV in Vietnam, a major pork-producing country in Asia. Through longitudinal surveillance at a central slaughterhouse in Hanoi, which sourced pigs from across the country, we found H1N1, H1N2, and H3N2 swIVs co-circulating. This finding is consistent with surveillance conducted in 2013–2014 (*13*,*26*) and with other studies in Vietnam (*23*,*27**,**28*), indicating that the swIV subtypes circulating in Vietnam are similar to those found across Asia and globally (*31*–*34*).

We found that the genetic diversity of swIV in Vietnam since 2010 is attributable to the persistence of several swine-origin H1N2 and H3N2 viruses, first reported in other countries in Asia and North America and likely imported via swine trade. We also identified repeat introductions of human pH1N1 viruses through reverse zoonosis. We found extensive reassortment of major swIV lineages, including H1N2 and H3N2 frequently acquiring pH1N1 internal genes and only rare acquisition of other swIV internal genes by pH1N1 lineage viruses. As a result, most recent swIVs from Vietnam contain pH1N1 internal genes. However, 2 divergent H1-δ virus lineages (originally derived from pre-2009 seasonal H1N1 viruses), detected in our most recent sample from 2019, maintained the TR lineage NA and internal genes (genotypes 10, 12, and 20) (Figure 3, panel B).

Although repeated introductions of pH1N1 into swine is consistent with previous swine surveillance from Vietnam (*13*) and southern China (*35*,*36*), the frequency of detections of new human pH1N1 virus lineages in swine in Vietnam has tapered off since 2015. Although onward transmission of pH1N1 was not sustained for most introductions, 1 lineage, first detected in northern Vietnam during 2013–2014 (*13*), persisted in swine for >4 years and reassorted with other prevailing swIV lineages. When introduced as components of reassortants, pH1N1-origin gene segments tend to be maintained in pigs (*27*), whereas viruses whose genomes are entirely derived from pH1N1 tend not to be sustained in the pig population. This phenomenon has been observed globally and in southern China and Hong Kong surveillance studies, which showed that viruses from purely pH1N1 failed to sustain the pig population after each introduction (*36*). Similar evidence of pH1N1 being sustained within swine populations has been reported from the United States (*37*) and Australia (*38*).

Pre-2009 seasonal H1N1 influenza-derived swine viruses (classified under H1-δ) are potentially gaining predominance in swine in Vietnam. Of note, an H1N2 H1-δ1a virus lineage with limited cross-reactivity to other H1 swIV lineages was detected in 2019, and serosurveillance showed evidence of circulation since March 2016. The increasing prevalence of H1-δ–like lineages increases the risk for zoonotic transmission. Previous studies demonstrated significant antigenic distance between pH1N1-like viruses and H1-δ cluster viruses (H1-δ–like and H1-δ1-a) (*29*,*30*), and current seasonal influenza vaccines do not elicit protection against H1-δ swIVs (39–*41*). It is therefore important to ascertain whether current diagnostics can distinguish pre-2009 H1 viruses from currently circulating human seasonal H1 strains.

H1-δ1a viruses likely entered Vietnam via imported swine, demonstrating the role of trade in the global dissemination of swIVs (*42*). East and Southeast Asia countries, including China, Thailand, and Vietnam, which produce >50% of pork globally, are hotspots for emerging infectious diseases from swine (*43*,*44*). Vietnam ranks second in Asia for pork production, producing 19.62 million heads in 2019 (*45*). In addition, pork consumption in Vietnam has risen rapidly, from 12.8 kg/head/year in 2001 to 31.4 kg/head/year in 2018 (*46*), mostly in the form of fresh pork. Vietnam has also been importing breeding hogs from the United States since 1996. On average, 553 breeding hogs were imported each year from the United States during the 2010s, with a peak in 2014–2015 (Appendix 2 Figure 2) (*47*). The H1-δ1a lineage may have been introduced to Vietnam by imported breeding hogs in early 2016, as was seen in mainland China in the early 1990s (*48*).

In each of the swIV lineages detected in Vietnam, we discovered evidence of localized circulation, as evidenced by periods of unsampled diversity and long phylogenetic branches, likely at the provincial level. Despite the limited mixing of swine populations in Vietnam before slaughter (*44*), the infrequent detection of persistent lineages in the central slaughterhouse suggests that the genetic diversity of swIV in Vietnam may be high. This diversity is likely driven by factors such as livestock density and turnover. In comparison, swine in the United States are exposed to a greater degree of mixing during their lifespan because they are transported across long distances for feeding and fattening, which results in replacement by advantageous swIV lineages (*37*).

Our findings indicate that the swIV gene pool in Vietnam is continually enriched by importations from North America and from other countries in Asia. Those viruses include a novel cluster of H1-δ1a (genotype 10) viruses, which may pose a zoonotic threat (*49*). As the H1-δ1a virus was found only during the last sample collection, its persistence is uncertain. Recurrent transmission of pH1N1 viruses from humans to swine and reassortment with other swIV lineages have increased genetic diversity. Hence, to limit further introductions and diversification of the swIV gene pool in Vietnam, it is important to actively monitor both local swine herds and imported swine.

Appendix 1 Sequence metadata and accession numbers for long-term epidemiology and evolution of swine influenza viruses, Vietnam.

Appendix 2 More information is available for long-term epidemiology and evolution of swine influenza viruses, Vietnam.
