# Supplementary material for: Long-Term Epidemiology and Evolution of Swine Influenza Viruses, Vietnam
Source: Emerg Infect Dis. 2023 Jul;29(7):1397–406. doi: 10.3201/eid2907.230165 (PMC10310380; doi:10.3201/eid2907.230165)
Supplement: Appendix 2 — More information is available for long-term epidemiology and evolution of swine influenza viruses, Vietnam. [file 23-0165-Techapp-s2.pdf]

Article DOI: http://doi.org/10.3201/eid2907.230165

EID cannot ensure accessibility for supplementary materials supplied by authors. Readers who have difficulty accessing supplementary content should contact the authors for assistance.

Long-term Epidemiology and Evolution of Swine Influenza Viruses, Vietnam

Appendix 2

Appendix 2 Table 1. Seroprevalence of antibodies against the swine H1-δ1a virus (A/swine/Hanoi/12–276/2019) in pigs in northern Vietnam.

Year	No. of swine sera tested by HAI assay	No. (%) seropositive*
2013	80	0 (0)
2014	120	0 (0)
2015	120	0 (0)
2016	120	12 (10)
2017	120	8 (6.7)
2018	120	4 (3.3)
2019	80	12 (15)
Total	760	36 (4.7)

*Seropositivity defined as HAI titer ≥40. HAI, hemagglutination inhibition.

Appendix 2 Table 2. HAI titers for individual H1-δ1a seropositive swine sera against other H1 SwIV lineages. Titers ≥40 are shown in bold.

Sera ID	Province of origin	Sampling Date	H1-δ1a A/swine/Hanoi/12–276/2019(H1N2)	H1-δ-like A/swine/Hanoi/11–260/2019(H1N2)	H1N1pdm09 A/California/04/2009 (H1N1)	H1-TR A/swine/Hanoi/7–305/2016(H1N2)
Ta_6_101M	Unknown	24/3/2016	**320**	20	<10	<10
Ta_6_667M	Unknown	21/7/2016	**160**	20	**160**	**40**
Ta_6_969M	Unknown	21/9/2016	**640**	**160**	**160**	**40**
Ta_7_119M	Unknown	27/10/2016	**640**	**320**	**40**	<10
Ta_7_120M	Unknown	27/10/2016	**80**	<10	10	<10
Ta_7_121M	Unknown	27/10/2016	**160**	20	**80**	**40**
Ta_7_301M	Ha Nam	25/11/2016	**80**	<10	**40**	<10
Ta_7_304M	Ha Noi	25/11/2016	**640**	**40**	10	<10
Ta_7_310M	Ha Nam	25/11/2016	**80**	10	<10	<10
Ta_7_313M	Ha Nam	25/11/2016	**160**	10	<10	<10
Ta_7_324M	Unknown	25/11/2016	**80**	**40**	**80**	**40**
Ta_7_422M	Unknown	28/12/2016	**80**	<10	<10	<10
Ta_7_721M	Ha Noi	23/2/2017	**640**	**40**	**40**	<10
Ta_7_871M	Ha Noi	23/3/2017	**80**	<10	20	<10
Ta_8_170M	Ha Noi	25/5/2017	**160**	<10	20	<10
Ta_8_321M	Thai Nguyen	22/6/2017	**160**	<10	<10	<10
Ta_8_477M	Ha Noi	26/7/2017	**160**	<10	**80**	20
Ta_8_627M	Ha Noi	24/8/2017	**160**	10	**80**	10
Ta_8_772M	Ha Nam	28/9/2017	**80**	<10	**80**	10
Ta_8_774M	Ha Nam	28/9/2017	**160**	<10	**40**	<10
Ta_10_129M	Hoa Binh	22/6/2018	**80**	**80**	<10	<10
Ta_10_578M	Ha Noi	27/9/2018	**320**	**320**	<10	<10
Ta_10_729M	Ha Noi	30/10/2018	**160**	**≥1280**	20	<10
Ta_10_875M	Ha Noi	27/11/2018	**80**	20	<10	<10
Ta_11_622M	Thai Binh	24/4/2019	**160**	**40**	10	10
Ta_11_631M	Thai Binh	24/4/2019	**80**	20	<10	<10
Ta_11_779M	Ha Noi	23/5/2019	**80**	**160**	<10	<10
Ta_11_928M	Thai Nguyen	25/6/2019	**≥1280**	**160**	**80**	10

Sera ID	Province of origin	Sampling Date	H1-δ1a A/swine/Hanoi/12–276/2019(H1N2)	H1-δ-like A/swine/Hanoi/11–260/2019(H1N2)	H1N1pdm09 A/California/04/2009 (H1N1)	H1-TR A/swine/Hanoi/7–305/2016(H1N2)
Ta_11_929M	Ha Noi	25/6/2019	**80**	20	20	<10
Ta_11_931M	Tuyen Quang	25/6/2019	**80**	10	10	<10
Ta_12_075M	Dong Nai	17/7/2019	**80**	**640**	<10	<10
Ta_12_079M	Dong Nai	17/7/2019	**80**	10	<10	<10
Ta_12_296M	Vinh Phuc	28/8/2019	**80**	20	20	<10
Ta_12_298M	Yen Bai	28/8/2019	**80**	**80**	<10	<10
Ta_12_300M	Yen Bai	28/8/2019	**80**	<10	10	<10
Ta_12_304M	Vinh Phuc	28/8/2019	**40**	**160**	10	<10

*HAI, hemagglutination inhibition; SwIV, swine influenza virus.

Appendix 2 Table 3. HAI titers for individual H1-TR seropositive swine sera against H1N1pdm09 antigen. Titers ≥40 are shown in bold.*

Sera ID	Sampling Date	H1N1pdm09 A/California/04/2009(H1N1)	H1-TR A/swine/Hanoi/7–305/2016 (H1N2)
Ta_1_121M	7/5/2013	**320**	**80**
Ta_1_211M	11/6/2013	**160**	**40**
Ta_1_637M	5/9/2013	**160**	**40**
Ta_1_847M	3/10/2013	**320**	**80**
Ta_2_276M	17/1/2014	**320**	**80**
Ta_2_536M	6/3/2014	**80**	**40**
Ta_3_234M	22/8/2014	**640**	**160**
Ta_4_001M	29/1/2015	**320**	**80**
Ta_4_417M	23/4/2015	**160**	**40**
Ta_5_618M	23/12/2015	**80**	**40**
Ta_6_667M	21/7/2016	**160**	**40**
Ta_6_969M	21/9/2016	**160**	**40**
Ta_7_121M	27/10/2016	**80**	**40**
Ta_7_324M	25/11/2016	**80**	**40**
Ta_7_426M	28/12/2016	**80**	**40**
Ta_8_174M	25/5/2017	**80**	**40**
Ta_8_622M	24/8/2017	**80**	**40**
Ta_9_973M	24/5/2018	**80**	**40**

*HAI, hemagglutination inhibition; TR, triple reassortant.

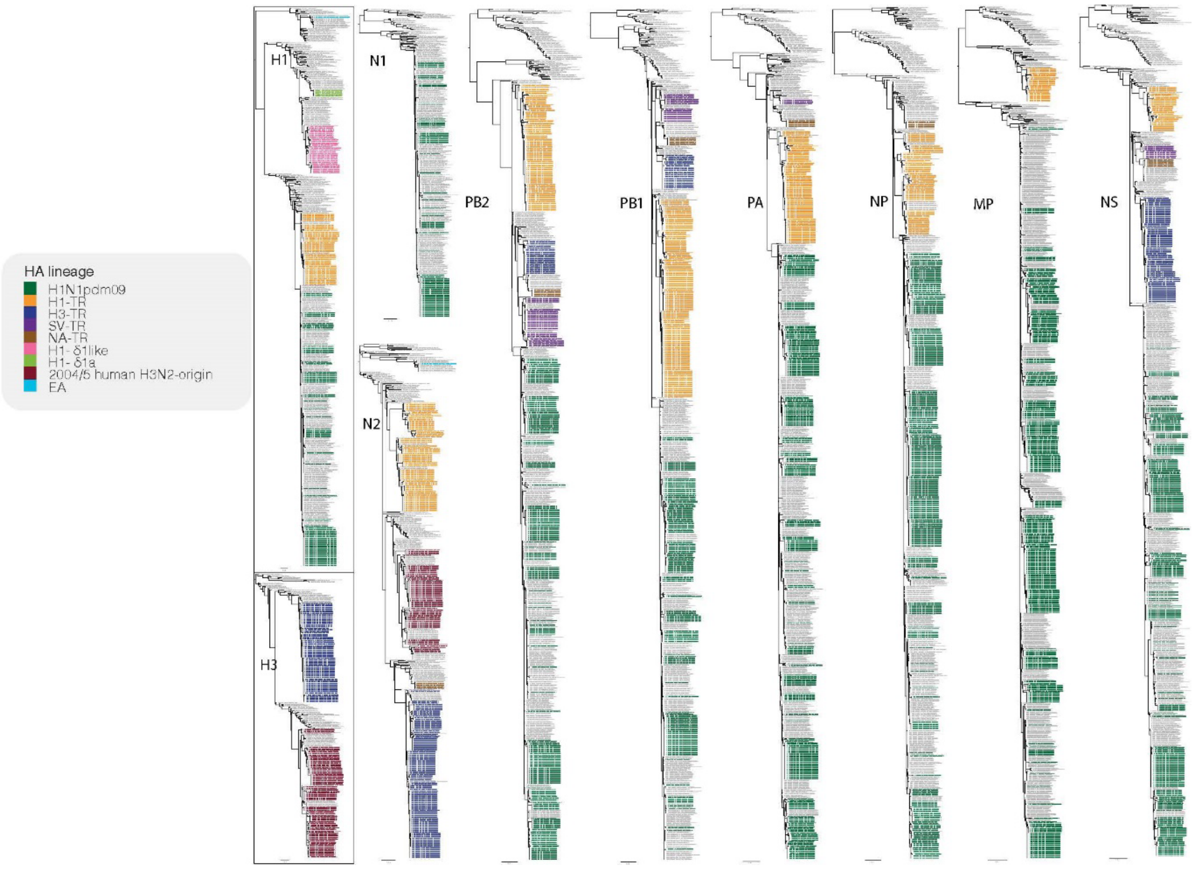

Appendix 2 Figure 1. Maximum likelihood phylogeny of each segment of SwIV in Vietnam. Maximum likelihood tree was constructed with IQ-TREE (http://www.iqtree.org/). Colors indicate HA lineage. Viruses from outside Vietnam are shown in gray. Isolates with no HA sequence are shown in pink. Scale bars represent nucleotide substitutions per site.

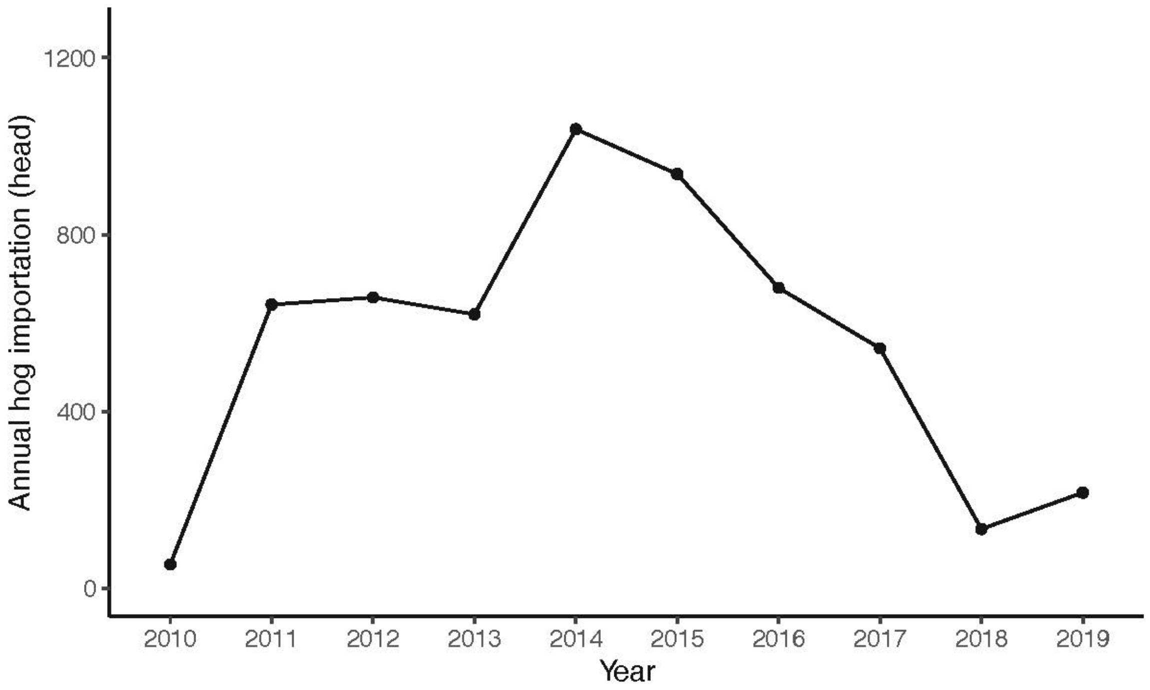

Appendix 2 Figure 2. Annual hog importation from United States to Vietnam in 2010–2019 (*47*).

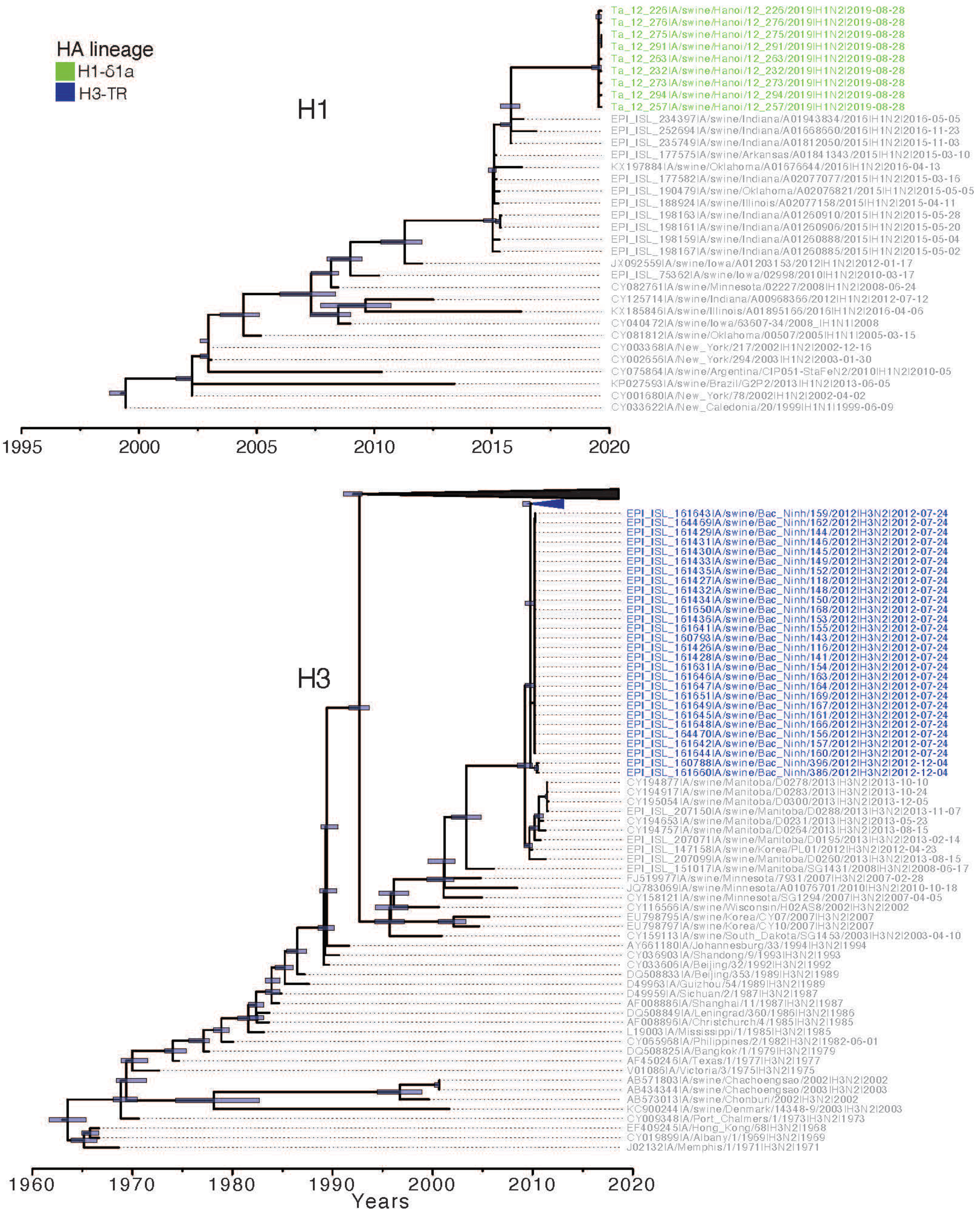

Appendix 2 Figure 3. The time-scaled phylogenetic tree of H1-δ1a and H3-TR using IQ-TREE v2.1.4, implementing a least square dating algorithm (LSD) (*21*). Blue bars displaying the 95% CI. Colors indicate HA lineage. Viruses from outside Vietnam are shown in gray.
